# Health, Security and Participation: A Structural Relationship Modeling among the Three Pillars of Active Ageing in China

**DOI:** 10.3390/ijerph17197255

**Published:** 2020-10-04

**Authors:** Yinan Yang, Yingying Meng, Pengtao Dong

**Affiliations:** 1 School of Public Administration, South China University of Technology, Guangzhou 510641, China; 2 Centre for Social Security Studies, Wuhan University, Wuhan 430072, China; 3 Policy Research Department, China National Committee on Ageing, Beijing 100011, China; dpt@cncaprc.gov.cn

**Keywords:** health, Healthy Ageing, latent variable, structural equation modeling

## Abstract

This paper explores and tests the impact of health and security on the participation of Chinese older people using data from the China Longitudinal Ageing Social Survey (CLASS) in 2014. Based on the framework of Active Ageing, the exogenous latent variables “health” and “security” are assumed to directly affect the endogenous latent variable “participation”, and indirectly affect it via mediating the function of “willingness”. The estimation results of the structural equation model show that health has a significant positive impact, while security has a significant negative impact on participation. In addition, health and security can significantly enhance the willingness of older people to participate. After the opposite effects of health and security are offset, their net effect on participation is generally negative. According to these empirical results, this paper concludes that the optimization of health coupled with the moderation of security level is more beneficial for promoting the participation of older people.

## 1. Introduction

A widely adopted strategy by most countries to meet the challenges from an ageing population is “Active Ageing” [1]. It was originated and developed from the concepts of “Successful Ageing”, “Healthy Ageing” and “Productive Ageing”. In 1997, at the G7 Summit Conference in Denver, “Active Ageing” was proposed for the first time, adding “security” to “health” and “participation”. The World Health Organization (WHO) formally defined Active Ageing as “the process of optimizing opportunities for health, security and participation to enhance the quality of life as people age” in 2002 [2]. Among the three pillars, as expressed in the WHO definition, “health” refers to physical, mental and social well-being; “security” addresses social, financial and physical security needs and rights to ensure the protection, safety and dignity of older people; and these support full “participation” in socioeconomic, cultural and spiritual activities to make a productive contribution to society in both paid and unpaid activities [3].

The WHO [4] especially stressed that “healthy older people are still valuable resources for family, peers, community and economic development and should be encouraged to continuously participate in social, economic, cultural and civic affairs”. The Madrid Political Declaration, issued by the Second World Congress on Ageing in 2002, states that “the potential of older people is a strong foundation for future development and can make the whole society better through their active participation” [5]. Especially for developing countries, it is still very important to give full play to the potential and surplus of older people [6]. Therefore, how to enhance the participation of older people has been a global academic focus and policy issue for decades. As a matter of fact, China officially declared itself as “actively responding to its ageing population” as early as 2006. Older people are still considered to be and highly praised as the creators of social wealth and active contributors to social development. In 2019, there were 254 million people over 60 years old and 176 million people over 65 years old. It is undoubtedly of great value to actively promote their social participation [7]. However, due to limited opportunities and resources to cultivate the younger generation’s employment and education, few effective ways have been found [8]. To further and better promote the participation of older Chinese people, one of the key issues worthy of exploration and clarification is the relationship, effect and paths between “security”, “health” and “participation” which have been regarded as merely side-by-side pillars of Active Ageing. 

Some empirical studies have examined the impact of “health” on “participation” but failed to reach a consistent conclusion on their relationship [9]. Many researchers have also examined the impact of social security on participation, and some of them have obtained positive effects, while others have found a negative effect [8,10]. Based on the WHO’s definition, “health”, “security” and “participation” are all multi-dimensional. Only by using multiple indicators can we capture the multi-dimensional profile of “Active Ageing” [11]. However, most of the current studies tend to focus on older people’s participation in economic activities by using a single manifest variable or examining it separately, though there are several indicators available. In order to accurately define and explain the internal relationship between the three pillars of Active Ageing, more indicators are needed to accurately reflect the participation of older Chinese people. Furthermore, more research is also needed to clarify the role of “health” and “security” in the Active Ageing process and to better understand the pathways of how “health” and “security” actually affect “participation”.

Based on the data of the China Longitudinal Ageing Social Survey (CLASS), this paper will quantitatively analyze the effect and impact mechanism of “health” and “security” on “participation” under the framework of Active Ageing. Compared with existing studies, the innovation of this paper lies in the following. First, three latent variables measured by multiple indicators are used for “health”, “security” and “participation”. In particular, three indicators, employment, voting and community and society service, are used to measure the economic, political and voluntary “participation” of older people. Second, a new mediating structural equation model is used by considering the mediating role of older people’s “willingness” in the impact of “health” and “security” on the endogenous outcome variable “participation”. The improvement in measurement and model design will contribute to a deeper and more accurate understanding of the structural relationships among these three pillars. Therefore, resource allocation and policy making could be made to be more beneficial and efficient to promote the participation of older Chinese people. Since 70% of all older people will live in developing countries by 2025, the experience of China will also benefit other countries.

## 2. Literature Review

In the 1980s, population ageing became a global phenomenon. International organizations, governments and multidisciplinary scholars began to explore the coping strategies for population ageing. Havighurst [12] first proposed the concept of “Successful Ageing”. Rowe and Kahn [13] explained and promoted the concept of “Successful Ageing”: no disease and disability and normal physical and mental function. This definition neglects the heterogeneity in physical function and important health influencing factors in older people. Its evaluation of “success” overemphasizes the dimension of physical health and has strong personal value judgment. In 1987, the WHO [14] put forward the concept of “Healthy Ageing” to pay more attention to the influencing factors in older people’s health. However, “Healthy Ageing” is a needs-based approach rather than an approach concerning the human rights of older people and still has the impression of “Active Ageing”. Butler [15] put forward the concept of “Productive Ageing”, which emphasizes the social participation of older people while mostly focusing on their participation in economic activities.

On the basis of “Successful Ageing”, “Healthy Ageing” and “Productive Ageing”, the WHO put forward the strategy of “Active Ageing” in 2002 [2]. In addition to “health” and “participation”, it added a dimension of “security”, formulated a three-pillar framework and extended “participation” from the economic field to social, cultural, spiritual and civic affairs fields and recognized the factors, in addition to health care, that affect how individuals and populations age. The OECD [16] defines “Active Ageing” as “the ability to lead productive activities in social and economic fields”. The EU [17] defines it as “being satisfied at work, remaining independent in life and participating more in society as citizens”. Zaidi et al. [11] interpreted “Active Ageing” as the situation in which “people can continue to participate in the normal labor market and other free productive labor and still be able to live a healthy, independent and secure life”. It is obvious that “participation” occupies a prominent position in Active Ageing [18].

Regarding the structural relationships among “health”, “security” and “participation”, Zaidi et al. [11] take the health and supporting environment as the basis of older people’s employment and social participation among the domains of the Active Ageing Index (AAI). Chen [8] and Song [19] also believe that health is the premise of Active Ageing, that participation is the core objective and that security is the supporting condition. If older people want to maintain meaningful productive activities, then they need continuous training at work and lifelong learning opportunities in the community [20]. Convenient and affordable public transportation should be provided for older people in both urban and rural areas, enabling them to participate fully in family and community life and political activities that affect their rights [21]. Miao, Hu and Gao [10] believe that there are interactions among the four influencing factors of positive psychology, physical health, social relations and social activity participation. Wu [7] pointed out that the optimal combination of health, security and participation is the key to achieving “Active Ageing”, but he did not explain how to combine the three. Xie and Wang [22] used the CLASS data in 2014 to classify older people into three types—high participation, low participation and family care—according to their participation in economic, social, political and family activities. However, they did not carry out further research to study the “participation” behavior of older people.

Based on the above theoretical speculation and analysis, a wealth of empirical research has been carried out to explore and test the structural relationships among “health”, “security” and “participation”. Tian [23] and Fang and Yi [24] investigated two phases of data from the China Health and Retirement Longitudinal Study (CHARLS) and found that health is an important factor that influences older people to continue to work, which was investigated through the use of five indicators: social health, general health and disease history, lifestyle and health behavior, physical dysfunction and mental and psychological health. Tong and Liao [25] used Chinese Longitudinal Healthy Longevity Survey (CHLHS) 2014 data and found that health status is positively correlated with older people’s participation, and the impact is heterogeneous when concerning urban and rural areas, gender and age. Deng et al. [26] used the data of the CHARLS 2011 and 2013 and found that self-rated health and self-care ability have a significant positive impact on older people’s labor participation, while chronic diseases and mental health have a negative impact.

Li [27] analyzed the economic activity participation of older people over 65 years of age by using 2010 Chinese Women Social Status Survey data and found that their economic participation was not high, and most of them mainly aimed to meet basic life needs. Social pension insurance also had a significant positive impact, while medical insurance had no significant effect. However, the explanatory variables were dummy variables (whether there is pension insurance), and the influence of pension benefits was not tested. The economic environment is positively linked to engagement in paid work, grandparental childcare and community and leisure activities, as examined by the China Health and Retirement Longitudinal Study [28]. Um, Zaidi and Choi [29] also found that, in Korea, a high employment rate among older workers can be attributed largely to the constraints of low pension income status.

Empirical research on other forms of older people’s participation, such as political participation and voluntary activities, is relatively rare. Liu et al. [30] examined older people’s participation in family production activities by using data from Beijing in 2000, 2006 and 2010 and found that the proportion of participation declined. They found that pension income and medical insurance had no significant impact, but the increase in social assistance income significantly reduced older people’s probability of doing housework. They explained that the older people receiving social assistance lacked the ability to do housework. Zhang [31] used the third-wave data of the Women Status Survey to investigate 422 individuals over 65 years of age in Zhejiang Province and found that their level of participation in elections was high, but their level of participation in decision-making was low. Health status was a prerequisite for political participation. 

In many studies, it has been found that willingness can play a mediating role in the participation behavior of older people. Based on the fourth survey data from the Living Conditions of the Chinese Urban and Rural Elders in 2015, Yu [32] found that good economic and health conditions promote the willingness of older people to participate in voluntary services, but they did not further test whether this “willingness” affected the final participation behavior of older people. A study in Taiwan found that somatosensory game interventions significantly increased the degree of social interaction and the maintenance of the willingness to exercise of old people living in nursing homes over time, and further significantly improved their happiness indicators [33]. Evidence from older Japanese people in residential facilities showed that their mental condition was positively related to the willingness to accept conversation and companionship from volunteers [34].

Generally, most of the current research focuses on older people’s participation in economic activities and rarely examines other activities such as political participation and community or social volunteer service. As mentioned above, “health”, “security” and “participation” are all multi-dimensional and multi-index measures. However, most of the existing studies use a single proxy variable or examine them separately, even though several indicators are available at times. It is hard to reflect on the overall profile of “health”, “security” and “participation” of older people in China, as well as the mechanism and effect among the three pillars.

## 3. Research Design

### 3.1. Data Source

The sample data used in this paper are from the CLASS, which is a continuous national random sampling survey that interviews older people over 60 years of age in each household. The first national baseline survey data from 2014 have been released publicly with a total of 11,510 interviewees. The mean age is 70, with a minimum age of 60 and a maximum age of 113. The demographic characteristics of this sample are demonstrated in Table 1.

### 3.2. Model Framework and Variable Measurement

**Figure 1:**
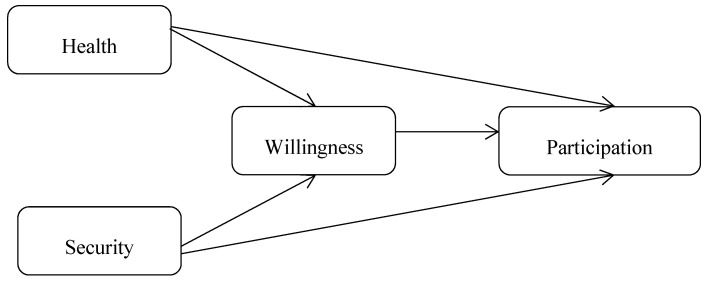
Theoretical structural relationship model for the three pillars of Active Ageing.

Based on the previous literature review, this paper proposes a structural relationship model for the three pillars of Active Ageing, as shown in Figure 1. 

In the model, health and security are independent variables and participation is an endogenous outcome variable. The mediating effect of “willingness” is also considered. Therefore, the influence of “health” and “security” on “participation” can be investigated and estimated. To reflect the multi-dimensional attributes and reduce measurement error, latent variables measured by multiple indicators are adopted for “health”, “security”, “willingness” and “participation”. “Participation” is measured by three indicators, as shown in Table 2, namely, participation in community voluntary activities (PCA), participation in paid work (PPW) and participation in local voting (PLV). “Health” is also measured by three indicators, which are self-rated health (SRH), activities of daily living (ADL) and self-care ability (SCA). “Security” is measured by three indicators, including social security income (SSI), social preferential treatment (SPT) and community activity facilities (CAF). “Willingness” is measured by four indicators, which are the willingness to participate in community affairs (WCA), willingness to serve society (WSS), willingness to learn new knowledge (WLK) and willingness to obtain useful information (WOI). These indicator variables and their measurement questions are shown in Table 2.

### 3.3. Models

Since “health”, “security”, “participation” and “willingness” are all latent variables, we use a structural equation model (SEM) to estimate their relationship. The structural equation model consists of two parts: the structural model reflecting the relationship between latent variables and the measurement model for measuring latent variables. The general matrix equation of the structural model is as follows:

(1) η=Bη+Γξ+ζ

It can be written for the model in Figure 1 as follows:

(2) $$\left[\begin{array}{l} \eta_{1}\\ \eta_{2} \end{array}\right]=\left(\begin{array}{cc} 0 & 0\\ \beta_{21} & 0 \end{array}\right)\left[\begin{array}{l} \eta_{1}\\ \eta_{2} \end{array}\right]+\left(\begin{array}{cc} \gamma_{11} & \gamma_{12}\\ \gamma_{21} & \gamma_{22} \end{array}\right)\left[\begin{array}{l} \xi_{1}\\ \xi_{2} \end{array}\right]+\left[\begin{array}{l} \zeta_{1}\\ \zeta_{2} \end{array}\right]$$

In Formula (2), *η*_1_ is the endogenous mediating variable “willingness”, *η*_2_ is the endogenous outcome variable “participation”, *β*_21_ represents the effect of “willingness” on “participation”, exogenous latent variables *ξ*_1_ and *ξ*_2_ are “health” and “security”, respectively, and *γ*_11_, *γ*_12_, *γ*_21_ and *γ*_22_ represent the effects of exogenous latent variables “health” and “security” on endogenous variables “willingness” and “participation”. *ζ*_1_ and *ζ*_2_ are the prediction errors of the two endogenous variables “willingness” and “participation”.

The matrix equation of the measurement model is as follows:

(3) $$Y=\Lambda_{y}\eta +\varepsilon$$

(4) $$X=\Lambda_{x}\xi +\delta$$

Formula (3) is the measurement model for the endogenous latent variables. Vector Y includes four indicators to measure the endogenous mediating variable “willingness”—WCA, WSS, WLK and WOI—and three indicators to measure the endogenous outcome variable “participation”—PCA, PPW and PLV. Λ*_y_* is the factor loading matrix and ε is the measurement error. Formula (4) is the measurement model for the exogenous latent variables. Vector X includes three indicators to measure “health”—SRH, ADL and SCA—and three indicators for measuring “security”—SSI, SPT and CAF. Λ_x_ is the factor loading matrix and *δ* is the measurement error.

## 4. Empirical Result Analysis

### 4.1. Descriptive Result Analysis

The descriptive statistical results of the indicators for the latent variables of health, security, willingness and participation are shown in Table 3.

**Figure 2:**
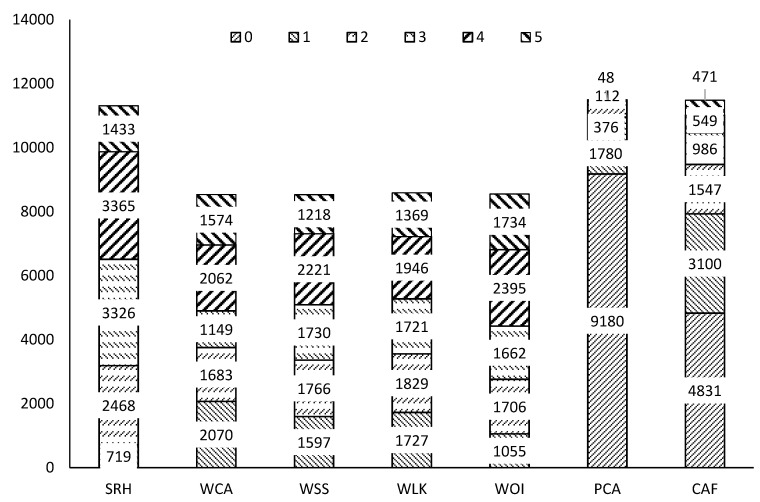
Column chart of six indicator variables. Note: SRH, self-rated health; WCA, willingness to participate in community affairs; WSS, willingness to serve society; WLK, willingness to learn new knowledge; WOI, willingness to obtain useful information; PCA, participation in community voluntary activities; CAF, community activity facilities.

The mean values of ADL, SCA and SRH in Table 3, reflecting the average level of physical function and self-care ability of older people, are much higher than those of other variables. Among the four indicator variables for “willingness”, the mean value of WOI is the highest. Among the “security” indicators, 29% of interviewed older people can obtain SPT; the average social security income (SSI) is RMB 1119/month, but there are still 9.56% of older people without any SSI, as shown in Figure 2. Of the communities, 42.07% do not provide any activity facilities for the older people, 26.99% provide one item, 13.47% provide two items and only 17.47% provide more than two items. Among the indicator variables for measuring “participation”, the proportion of older people engaged in paid work is 19%, and the proportion who have participated in voting elections is 46%. Although the indicator PCA covers a wide range of community voluntary activities, its mean value is only 0.27. It can also be seen from Figure 2 that 9180 people did not participate in voluntary activities, accounting for 79.85% of the sample, 15.48% participated in one activity and only 4.66% participated in more than two activities, which demonstrates that the participation rate in voluntary activities for older people is quite low.

### 4.2. Base Model Estimation Results

The maximum likelihood (ML) estimation is the default method used by the statistical software Stata15.0 (StataCorpLLC, College Station, TX, USA) for SEM. In the case of large samples, even if the measurement variables do not obey a multivariate normal distribution, the ML estimators will still be unbiased and asymptotically efficient [35,36]. Therefore, we use the ML method to estimate SEM. Considering that there are three binary variables in the measurement model, we also report the Satorra–Bentler standard error. The Satorra–Bentler standard error is more robust and efficient when the measurement variables are not normal [37]. The results are shown in Table 4.

According to the estimated results in Table 4, the factor loadings of the measurement variables for the four latent variables are all significant at the 1% level, which indicates that the measurement model is validly accepted. Health, willingness and security can explain 33.2% of the variance in participation. The R^2^ coefficient of the whole model is 0.969. Comparing models (1) and (2), it can be seen that the parameter estimators obtained by the ML method are the same, but the standard error is different. The Satorra–Bentler standard error is lower, the corresponding z values are larger and the significance level is higher. Henceforth, we choose to report the Satorra–Bentler standard error estimators.

From the estimation coefficient, health has a 1% level significant positive effect on older people’s “participation”, which is measured by paid work, local affairs voting and community volunteer activities. For every unit of health improvement, older people’s participation increases by 11.98%; health also has a 1% level significant positive effect on older people’s “willingness” to participate. For every unit of health improvement, “willingness” to participate increases by 63.15%, and “willingness” has a significant promotion effect on “participation” at the 1% level. This shows that health can not only promote the “participation” behavior of older people but also enhance their “willingness” to participate, which then continues to promote actual participation actions.

“Security” has a 1% level significant negative impact on “participation”. The “security” of older people increases by one unit, and the “participation” of older people decreases by 0.01%. Although the negative coefficient is quite small, it should not be overlooked. The three indicators of “security” are SSI, social preferential treatment and community facilities for the aged, which are biased toward “material security”. The negative influence coefficient means that the level of material security should not be too high. We should not take the optimization of “security” as the policy orientation but rather adhere to the “moderate” security level. A study in the United States shows that economic factors are the most important motivation for older people to participate in employment after retirement [38,39]. Chinese scholars have found that the main motivation of most older people returning to work is to increase their income, while retirees with good economic conditions are less willing to return to work [40]. Li [27] also found that the participation of older people is mostly passive, just to meet the basic needs of life.

The estimation results in Table 4 show that health and security have a significant positive impact on “willingness”, while “willingness” has a significant positive impact on “participation”. This finding shows that better health and a high level of security can promote the participation behavior of older people by indirectly enhancing their “willingness”. Yu [32] found that good economic and health conditions can promote older people’s willingness to participate in voluntary services, but the author did not test whether “willingness” affects the participation behavior of older people.

To obtain the total (net) effects of “health”, “security” and “willingness” on “participation”, we report the standardized coefficients in a path figure, as shown below.

**Figure 3:**
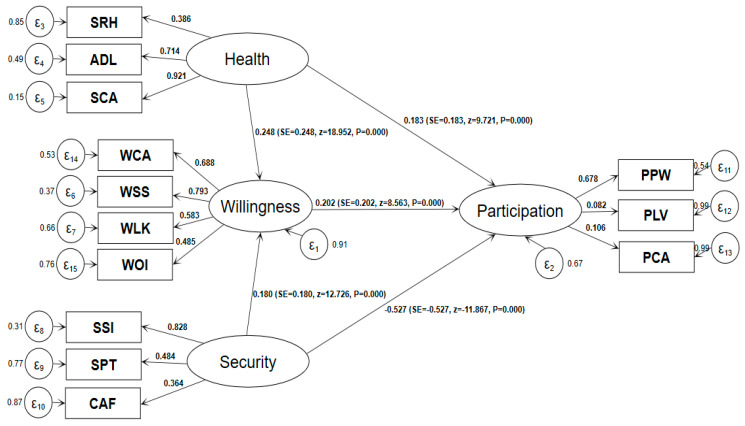
Model estimation results (standardization coefficient). Note: Maximum likelihood estimation, Satorra–Bentler standard error; N = 8061; ll = −201259.01; R^2^ = 0.969; SRMR = 0.072.

According to the standardized coefficients in Figure 3, health has both a direct positive effect (0.183) and an indirect positive effect on participation, namely, health→willingness→participation (0.248 × 0.202 = 0.05), and the sum of the two is 0.233. Security has both a direct negative effect (−0.527) and an indirect positive effect on participation, namely, security→willingness→participation (0.18 × 0.202 = 0.036), and the sum of the two is −0.491. As both of them are standardized coefficients, the negative effect of security on participation is greater than that of health’s positive effect on participation. As for as the data of the CLASS 2014 used in this paper, after the effects of health and security are offset, their net effect on older people’s “participation” is generally negative.

### 4.3. Multiple-Group Comparisons Using SEM

#### 4.3.1. Age Cohorts

Compared with young people, the health status of older people is very heterogeneous. Some older people are disabled, while some of them still have the physical and mental abilities of young people [4]. The individual diversity of older people also tends to increase with age [2]. To investigate the heterogeneity of older people’s “participation”, we used a multi-group comparison of the SEM. We divided older people into five age groups: 60–64, 65–69, 70–74, 75–79 and over 80 years old. To reduce the impact of missing values, we use the ML with missing values (MLMV) method, which is a full information estimation method that does not delete any missing observations by using a mixed log likelihood fitting function according to the types of missing data [35,41]. The estimated results are as follows.

In Table 4, only 8061 observations were estimated by the ML method, while in Table 5, 11,510 individual observations were estimated by using the MLMV method. This improvement can much more thoroughly extract the information contained in the sample and improve the quality of the statistical inference.

It can be seen from the estimation results in Table 5 that the influence of health on the “participation” of older people is significantly positive at the level of 1% in all five age groups, and the promotion effect of health on “willingness” increases with age up to the group of 80 years of age and above. This finding shows that as long as health conditions permit it, the “willingness” and behavior of older people remain active, which fully illustrates the basic premise of the role of health in older people’s participation. The impact of security on “participation” is still significantly negative in the five age groups, which indicates that the higher the level of material security, such as income, the lower the participation of older people of all ages. The influence of security on “willingness” is significantly positive in the first three age groups, but it is not significant in the 75–79 and 80 years of age and above groups. This finding shows that older people over 75 years of age are more inclined to enjoy their remaining lifetime, and improving economic and material security treatment will no longer enhance their willingness to participate. The influence coefficient of “willingness” on “participation” gradually decreases in the first four age groups and is not significant after the age of 80, which indicates that the possibility of a change in willingness to participate decreases with age and that older people have willingness but decreased physical capacity as they become older.

#### 4.3.2. Groups by Gender

Due to the longer life expectancy of women, ageing is even called the feminization process [2]. Women are more likely to suffer from poverty, disability and social isolation in their old age than are men. To investigate gender heterogeneity in the participation of older people, we also used a structural equation model to estimate gender differences. The estimation method is still the MLMV method, and robust standard errors are reported. The estimated results are shown in Table 6.

It can be seen from Table 6 that, compared with women, health has a greater impact on the “willingness” and “participation” of male older people, while “security” has a lesser impact on the “willingness” and “participation” of male older people. This finding shows that the participation of male older people is more dependent on “health”, while the “participation” of female older people is more dependent on economic and material “security” conditions. The effect of “willingness” on men is greater than that on women, which indicates that the “willingness” of male older people is transformed into “participation” behavior to a greater extent. As the heads of households, men have a higher degree of participation for a longer time, as long as their health conditions permit. Female older people need to take care of their grandchildren, do housework and so on. They pay more attention to the attractiveness of economic and material conditions, and their “willingness” is less likely to become final “participation” actions.

## 5. Discussion

The old age period is an important stage of life in which people can still do things, progress and have fun. However, society has a proportion of older people who remain active. Under the instruction of the Active Ageing strategy, it is undoubtedly of great theoretical and practical value to make more suitable overall arrangements for China’s health, security and participation policies. Quantitative evidence from empirical research can provide more detailed guidance for public health and ageing policy formulation and reform. 

From the empirical results in Figure 3, we can see that the influence coefficients of “security” on the “willingness” and “participation” of older people are opposites, indicating that improving the level of economic and material security helps enhance older people’s willingness to participate, but the impact on actual participation is negative. This finding shows that the economic and material security factor is only one aspect of the reason for older people to consider “participation”, and other conditions such as social justice, crisis rescue, prevention of abuse and protection of rights and interests are also be considered in decision-making. Since the participation rate of older people in China is indeed low, as described in Table 2 and Li’s [27] research results, it is not enough to only pay attention to the economic, material, facilities and other “difficult” aspects of security for older people’s “participation”. More attention should also be paid to spiritual, cultural and legal protection, such as human resource planning, education and training, employment policy, rights and interests protection and hobby cultivation, to further transform the “willingness” of older people to participate in final participation behavior.

This article also reveals that we need to further improve the health level of older people. According to the Health China Action Promotion Committee, in 2018, China’s average life expectancy was 77 years, of which 68.7 years were expected to be healthy life years. For approximately 8.3 years, older people live with diseases, including 40 million disabled older people and 20 million completely disabled older people. Another 180 million older people with one or more chronic diseases account for 75% of the older population. In 2016, the World Health Organization [4] defined “Healthy Ageing” in its Global Report on Ageing and Health as “the process of developing and maintaining the functions required for healthy life of the elderly” based on the perspective of “function”. This definition mainly depends on older people’s internal abilities, supportive environment and their interaction. To prolong the healthy life expectancy of older people and enhance their self-care ability and function, we should take various measures to improve their internal ability and supportive environment. Our research suggests we should adhere to the appropriate level of economic and material security and vigorously develop spiritual, cultural, social, legal and social organizations and other nonmaterial security content to turn the “willingness" of older people into actual participation behavior.

From the grouping point of view, the influence of health on older people’s “participation” is significantly positive in all five age groups, and the promotion effect on “willingness” increases with age until 80 years of age or older. The influence of security on “participation” is still significantly negative in the five age groups, and the influence of security on “willingness” is significantly positive in the first three age groups but no longer significant in the two groups aged 75–79 and 80 years and above. From the perspective of gender, male older people’s “participation” is more dependent on “health”, while female older people’s “participation” is more dependent on economic and material “security”. Women fare worse than men in most countries [2,11]. In China, female older people need to take care of their grandchildren, do housework and so on. This unpaid contribution to the family would explain why they pay more attention to the attractiveness of economic and material conditions and their “willingness” is less likely to become final “participation” actions compared with older men.

Since the main objective of this study is to explore the correlation among health, security and participation, therefore, the endogenous problems caused by the reverse causality of health and participation are not fully discussed and dealt with in the model. Another issue to consider is that Health Ageing has replaced the World Health Organization’s previous Active Ageing policy framework. Health Ageing, like Active Ageing, emphasizes the need for action across multiple sectors and enabling older people to remain a resource to their families, communities and economies. Different from Active Ageing, Health Ageing emphasizes the role of intrinsic capacities and external supportive environments [4]. Accordingly, further research is needed to explore the relationship between health, security and elderly participation in these new dimensions.

## 6. Conclusions

Based on the empirical results from the data of the CLASS in 2014, this paper finds that “Health” can enhance not only older people’s “participation” behavior but also their “willingness” to participate and ultimately promote their actual participation; “security” significantly positively affects the “willingness” of older people and then further positively affects their “participation”, but the direct impact of “security” on “participation” is significantly negative. Compared with the standardization coefficient, it can be seen that after the impacts of “health” and “security” are offset, their net effect on “participation” is negative. Based on these empirical results, it can be concluded that the optimization of “health”, coupled with the moderation of economic and material “security” level, is more beneficial to promoting the participation of older people, which is inconsistent with the viewpoint of maximizing “health” and “security” to promote “participation”, as declared by the WHO.

The following policy proposals could be suggested: first, China’s public health policy can further focus on the optimal process of older people’s health, further extend their healthy life expectancy and enhance their ability of self-care and function; second, the economic and material security policy for older people must adhere to the “moderate” principle, not the “optimal” principle. In addition, more attention should be paid to the development of non-material supportive environments, including the physical, spiritual, cultural, social and legal security content; third, relatively young older people make up the key group to promote participation and explore the value of human resources; and fourth, economic incentives and material security can mainly be used to promote the participation of female older people, while the “participation” of male older people should pay more attention to improving or maintaining their health status and physical function. There is also a need for an emphasis on reducing gender disparity in the experiences of Active Ageing.

## Figures and Tables

**Table 1:** Demographic characteristics of the samples.

Variables	Frequency	Percentage (%)
Gender		
male	5528	48.02%
female	5983	51.98
Age		
60–64	3616	31.42%
65–69	2399	20.84%
70–74	1950	16.94%
75–79	1690	14.68%
80–84	1241	10.78%
85+	614	5.34%
Marital status		
married	7449	64.79%
widowed	3855	33.53
divorced	114	0.99%
unmarried	80	0.7%
Education		
below primary school	3949	34.33%
primary school	3545	30.82%
high school	3331	28.95
college and higher	679	5.90%
Identity		
rural	5963	51.85%
non-rural	5537	48.15%

**Table 2:** Indicators of the latent variables and their measurement questions.

Latent Variables	Indicator Variables and Their Measurement Questions
Health	SRH, self-rated health. The interviewed older people are asked what they think of their current health. The answer options are: very unhealthy, relatively unhealthy, general, relatively healthy or very healthy, with the values of 1, 2, 3, 4 or 5, respectively. This indicator is an ordered categorical variable. ADL, activities of daily living. Eleven questions are asked, including the following: can you make a call, tidy up, get dressed, take a bath, eat, take medicine, have urinary incontinence, have fecal incontinence, go to the toilet, move to the bed or chair and walk indoors? The answer options are: totally unable, need some help and need no help from others, which are assigned values of 1, 2 and 3, respectively; then, the 11 variables are summed to obtain the total score. It is a numerical variable. SCA, self-care ability. Nine questions were asked, including the following: can you go up and down stairs, have you ever fallen down, can you walk outside, take public transport, shop, manage your money, lift 10 kg, cook and do housework? The answer options are: cannot do it at all, need some help and do not need help from others, assigned values of 1, 2 and 3, respectively; then, the nine variables are summed to obtain the total score. It is a numerical variable.
Security	SSI, social security income, including pension benefits for urban employees, pension benefits for urban residents, pension benefits for rural residents, social assistant benefits, advanced age allowance, home-based endowment service subsidy, one-child family subsidy and other government assistance. We sum these ten kinds of benefits to obtain the social security income of older people. It is a numerical variable. SPT, social preferential treatment for older people, including free bus passes, park tours and so on. The answer options are yes or no, which are assigned values of 0 or 1, respectively. It is a dummy variable. CAF, community activity facilities. Do you have any of the following venues or facilities in your community: aged activity room, fitness room, chess and card room, library, outdoor activity venue? The answer options are “yes” or “no”, which are assigned values of 1 or 0, respectively. The seven items are summed, and values of 0, 1, 2, 3, 4, 5 and 6 are obtained. Because there are only nine individuals with a value of 6, they are merged into 5. It is a numerical variable.
Willingness	The questionnaire asked “do you think the following description is in line with your current situation?”: willingness to participate in community affairs, WCA; serve society, WSS; like to learn new knowledge, WLK; and obtain useful information, WOI. The answer options for these four questions are completely inconsistent, relatively inconsistent, general, relatively consistent and completely consistent. We set them as four ordinal variables with values of 1, 2, 3, 4 and 5. All three indicators are ordered categorical variables.
Participation	PCA, participate in community voluntary activities. Older people are asked if they participated in the following eight activities in the past three months: community security patrol, caring for other older people, environmental protection, dispute resolution, accompanying chat, professional service or taking care of a neighbor’s children. The answer options for these questions are as follows: have participated in or never participated in, which are assigned values of 1 or 0, respectively. The answer results of the eight questions are summed up, and 0, 1, 2, 3, 4, 5 and 6 are obtained. The value of 0 stands for not participating in any of the above eight activities in the previous three months, and the other values stand for participating in 1, 2, 3, 4, 5 and 6 activities. Since there are only 10 and three individuals with values of 5 and 6, respectively, we combine the values of 5 and 6 into the category of 4. This indicator is an ordered categorical variable. PPW, participate in paid work. Are you currently engaged in paid work or activities? The answer options are yes or no, assigned as 1 or 0, respectively. It is a dummy variable. PLV, participate in local voting. Have you participated in the voting election of local residents’ committee or villagers’ committee in the past three years? The answer options are yes or no, which are assigned as 1 or 0, respectively. It is a dummy variable.

**Table 3:** Descriptive statistical results of indicator variables.

Variables	N	Mean	SD	Min	Max
ADL	11,377	31.93	2.690	11	33
SCA	11,281	25.24	3.290	11	27
SRH	11,311	3.210	1.110	1	5
WCA	8538	2.930	1.460	1	5
WSS	8532	2.960	1.340	1	5
WLK	8592	2.930	1.370	1	5
WOI	8552	3.240	1.310	1	5
PCA	11,496	0.270	0.610	0	4
PLV	11,488	0.460	0.500	0	1
PPW	11,503	0.190	0.390	0	1
SPT	11,479	0.290	0.460	0	1
CAF	11,484	1.190	1.450	0	5
SSI	11,511	1119	1434	0	14,400

**Table 4:** Model maximum likelihood estimation results.

Model		(1)	(2)
Standard Error		OIM	Satorra–Bentler
**A. Structural model**			
	Health→Participation	0.1198 *** (10.14)	0.1198 *** (14.58)
	Health→Willingness	0.6315 *** (16.53)	0.6315 *** (16.87)
	Security→Participation	−0.0001 *** (−16.53)	−0.0001 *** (−22.14)
	Security→Willingness	0.0002 *** (11.62)	0.0002 *** (11.87)
	Willingness→Participation	0.0518 *** (7.31)	0.0518 *** (10.37)
**B. Measurement model**			
Participation	PPW	1.000 (.)	1.000 (.)
	cons	0.1995 *** (44.65)	0.1995 *** (44.82)
	PLV	0.1501 *** (4.09)	0.1501 *** (4.73)
	cons	0.4929 *** (88.51)	0.4929 *** (88.51)
	PCA	0.2526 ** (2.20)	0.2526 *** (5.48)
	cons	0.2960 *** (41.07)	0.2960 *** (41.08)
Health	SRH	1.000 (.)	1.000 (.)
	cons	3.3290 *** (277.91)	3.3290 *** (277.90)
	ADL	2.6902 *** (32.17)	2.6902 *** (17.93)
	cons	32.4645 *** (1862.10)	32.4645 *** (1861.98)
	SCA	5.2608 *** (28.59)	5.2608 *** (26.60)
	cons	25.9187 *** (980.61)	25.9187 *** (980.55)
Willingness	WSS	1.000 (.)	1.000 (.)
	cons	2.9728 *** (199.88)	2.9728 *** (199.47)
	WCA	0.9473 *** (55.67)	0.9473 *** (45.84)
	cons	2.9340 *** (180.55)	2.9340 *** (180.27)
	WLK	0.7542 *** (34.25)	0.7542 *** (45.28)
	cons	2.9412 *** (192.77)	2.9412 *** (192.55)
	WOI	0.6009 *** (28.87)	0.6009 *** (36.67)
	cons	3.2569 *** (223.04)	3.2569 *** (222.85)
Security	SSI	1.000 (.)	1.000 (.)
	cons	1335.6962 *** (79.89)	1335.6962 *** (79.89)
	SPT	0.0002 *** (21.89)	0.0002 *** (26.38)
	cons	0.3292 *** (62.90)	0.3292 *** (62.90)
	CAF	0.0004 *** (19.67)	0.0004 *** (22.67)
	cons	1.3208 *** (82.06)	1.3208 *** (82.06)
**C. Goodness-of-fit indices**		N = 8061; ll = −201259.01; SRMR = 0.072; R^2^_Willingness _= 0.094; R^2^_Participation_ = 0.332; R^2^_total_ = 0.969	

Note: (1)The factor loading of the first indicator for each latent variable is set to 1 by default, the z values are in brackets and ***, **, * represent 1%, 5% and 10% significance, respectively; (2) SRH, self-rated health; ADL, activities of daily living; SCA, self-care ability; SSI, social security income; SPT, social preferential treatment for older people; CAF, community activity facilities; WCA, willingness to participate in community affairs; WSS, serve society; WLK, like to learn new knowledge; WOI, obtain useful information; PCA, participate in community voluntary activities; PPW, participate in paid work; PLV, participate in local affairs voting.

**Table 5:** Multi-group comparison by age cohort (N = 11,510).

Model	(3)	(4)	(5)	(6)	(7)
Age Cohort	60–64	65–69	70–74	75–79	80+
**A. Structural Model**					
Health→Participation	0.1598 *** (6.77)	0.0748 *** (4.11)	0.1049 *** (4.88)	0.1045 *** (4.45)	0.1145 *** (4.82)
Health→Willingness	0.3493 *** (2.98)	0.5992 *** (4.43)	0.7331 *** (5.83)	0.9595 *** (8.69)	0.6869 *** (10.67)
Security→Participation	−0.0002 *** (−7.88)	−0.0001 *** (−6.51)	−0.0001 *** (−8.18)	−0.0001 *** (−6.51)	−0.0001 *** (−4.61)
Security→Willingness	0.0005 *** (7.18)	0.0003 *** (8.35)	0.0001 *** (4.09)	0.0000 (0.18)	−0.0001 (−1.55)
Willingness→Participation	0.1082 *** (5.03)	0.0715 *** (3.37)	0.0439 *** (2.64)	0.0236 ** (1.99)	0.0093 (1.08)
**B. Measurement model**					
N	3616	2399	1950	1690	1855
R^2^	0.977	0.963	0.965	0.972	0.972

**Note**: Parameter estimation method: Maximum likelihood with missing values (MLMV), robust standard error, z value in brackets, ***, ** and * represent 1%, 5% and 10% significance, respectively.

**Table 6:** Multi-group comparison by gender (N = 11,510).

Model	(8)	(9)
Gender cohort	Female	Male
**A. Structural model**		
Health→Participation	0.0693 *** (6.48)	0.1068 *** (8.13)
Health→Willingness	0.6626 *** (12.33)	0.7429 *** (11.10)
Security→Participation	−0.0000 *** (−8.61)	−0.0001 *** (−9.43)
Security→Willingness	0.0002 *** (8.93)	0.0001 *** (7.58)
Willingness→Participation	0.0267 *** (3.31)	0.0515 *** (4.35)
**B. Measurement model**		
N	5945	5479
R^2^	0.993	0.988

Note: Parameter estimation method: MLMV, robust standard error, z value in brackets, ***, ** and * represent 1%, 5% and 10% significance, respectively.
